# Positive health coaching: a conceptual analysis

**DOI:** 10.3389/fpsyg.2025.1597867

**Published:** 2025-07-18

**Authors:** Croía Loughnane, Jolanta Burke, Elaine Byrne, Marina Iglesias-Cans, Ciara Scott, Mary Collins, Roger Bretherton, Roisin O’Donovan, Christian van Nieuwerburgh, Pádraic J. Dunne

**Affiliations:** Centre for Positive Health Sciences, Royal College of Surgeons in Ireland (RCSI), University of Medicine and Health Sciences, Dublin, Ireland

**Keywords:** positive health, health coaching, health psychology, positive psychology, lifestyle medicine, health promotion, disease prevention

## Abstract

Positive health coaching (PHC) is a novel approach designed to address the growing need for a more holistic perspective on health to combat the rising epidemic of non-communicable diseases (NCD). Extending beyond disease prevention, PHC promotes complete physical, mental, and social wellbeing, aligning with the World Health Organisation (WHO) vision of health as a resource for everyday life. This paper introduces the conceptual analysis for PHC, which emerged from the wider theoretical base of Positive Health Sciences (PHS). PHC is an interdisciplinary field that integrates medical and psychological approaches to promote a dynamic and comprehensive orientation and journey toward positive health. PHC bridges the gap between theory and practice by integrating insights and strategies from lifestyle medicine, positive psychology, health psychology, and coaching psychology. While the first three elements establish the PHS foundation of PHC, the fourth component operationalizes these theories through coaching strategies. This paper aims to guide readers on a journey from understanding the foundational principles of PHS to exploring how PHC operationalizes these concepts to promote overall health and wellbeing.

## 1 Introduction

**Figure 1:**
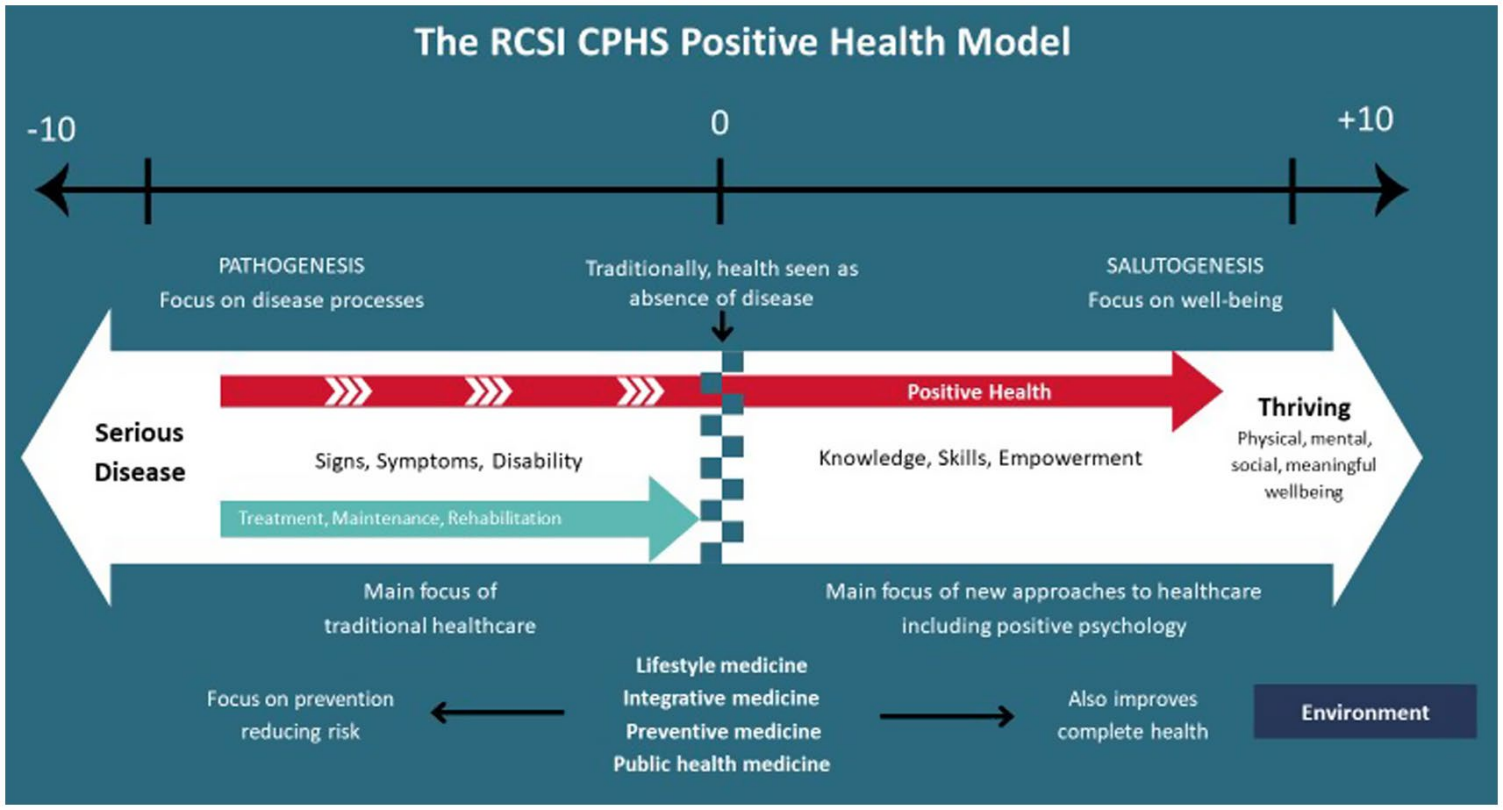
Positive health model proposed by the RCSI Centre for Positive Health Sciences, adapted with permission from O'Boyle et al. (2023), RCSI Centre for Positive Health Sciences.

Most Western healthcare systems are geared more toward treating illness and disease rather than preventing them or optimizing overall wellbeing. This is especially true with the rising burden of non-communicable diseases (NCDs), which represent 75% of all deaths globally (World Health Organisation, 2024). These systems have traditionally focused on curing or rehabilitating patients, preventing further deterioration, and improving quality of life, especially for those with chronic conditions. While crucial for treating disease and extending the lives of millions, this approach offers limited value for those not suffering from an illness but not flourishing either. These citizens can fall through the cracks in society, becoming the worried well, never completely feeling content or completely healthy. Likewise, this approach does not acknowledge the possibility of flourishing among those at risk of or living with chronic diseases. This highlights the need for a holistic view of health that encompasses all aspects of health and wellbeing. Positive Health Coaching (PHC) seeks to address this gap by combining evidence-based interventions with practical coaching strategies to support individuals to flourish (Figure 1).

**Figure 2:**
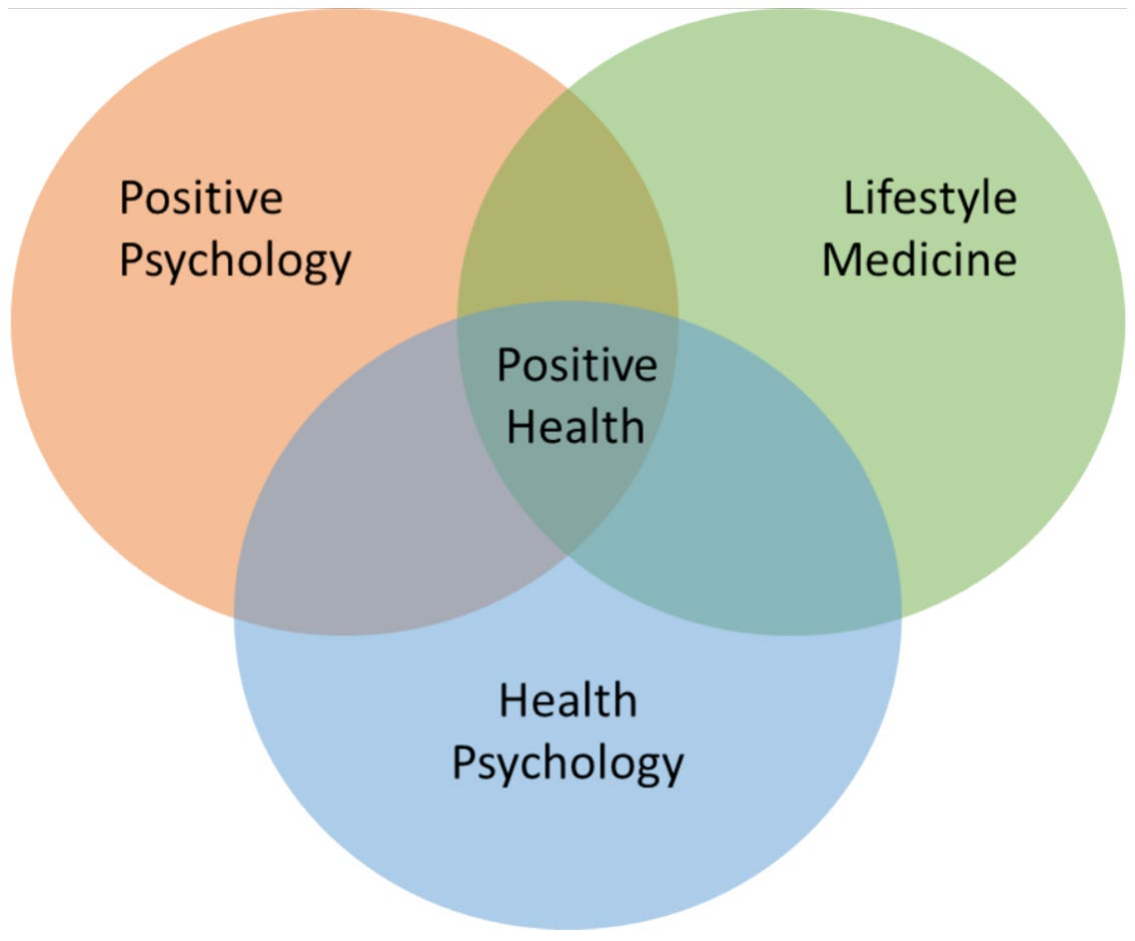
Positive health sciences paradigm. The positive health paradigm integrates the existing fields of positive psychology, lifestyle medicine and health psychology to provide a foundational theory of whole health and evidence-based interventions.

Positive Health Sciences (PHS) provides the theoretical foundation for PHC, emphasizing the integration of medical and psychological approaches to foster a more rigorous and holistic approach to health and wellbeing. Rooted in positive psychology (Seligman and Csikszentmihalyi, 2000), lifestyle medicine (Egger et al., 2009), health psychology (Krantz et al., 1985), and coaching psychology, this interdisciplinary field integrates medical and psychological approaches to promote a dynamic and comprehensive journey toward positive health (Figures 1, 2). By uniting the “mind” and the “body,” PHS enables professionals to view individuals as whole persons rather than fragmented parts. This field incorporates various evidence-based interventions to foster both eudemonic (realizing personal potential) and hedonic (pleasure and satisfaction) wellbeing, along with physiological indicators of health (i.e., cardiovascular, neuroendocrine, and immunological parameters) and lifestyle factors (adequate sleep, regular physical activity, healthy eating, stress management, avoiding alcohol and tobacco as well as cultivating positive relationships) that are essential for human functioning (Ryff and Singer, 2008; ACLM, 2022).

This paper analyses each concept of PHS, followed by the role of coaching in translating these concepts into practice. We then introduce PHC and how this new coaching approach weaves the theoretical concepts and coaching practices together to create a more holistic approach to health and wellbeing. By doing so, we aim to guide researchers and practitioners from healthcare, psychology, and coaching backgrounds by identifying the core elements required to understand and practice PHC. Furthermore, we seek to inform policies and curricula for PHC, emphasizing the importance of integrating evidence-based approaches into practice and training.

## 2 Positive health sciences

### 2.1 Lifestyle medicine: the foundations for positive health

Lifestyle medicine is an evidence-based clinical approach focused on combatting the rise of NCDs through health promotion, disease prevention, and management of chronic illnesses through therapeutic interventions targeting six foundational pillars: nutrition, physical activity, sleep, stress management, positive social connections, and the avoidance of risky substances (ACLM, 2022). Lifestyle medicine works to treat the whole person by employing lifestyle changes as its foundation for health (Lippman et al., 2024). Lifestyle medicine is predominantly delivered by clinicians trained and certified in this speciality to prevent, treat, and often reverse chronic disease (Collings et al., 2022; Frates, 2019; O'Boyle et al., 2023; Lippman et al., 2024). Grounded in robust evidence, lifestyle medicine emphasizes how daily lifestyle choices directly influence health outcomes, particularly relating to chronic diseases like cardiovascular disease, diabetes, and certain cancers (Doughty et al., 2017; Bodai et al., 2018). While the role of lifestyle in health and wellbeing has been evident for thousands of years, lifestyle medicine provides an influential shift in healthcare from the traditional biomedical view to a more holistic biopsychosocial approach to health (Doughty et al., 2017).

The pillars of lifestyle medicine serve as a foundation for PHS, guiding its focus on empowering individuals to make sustainable lifestyle changes that promote resilience and flourishing. The pillars of lifestyle provide a holistic view of a person’s physical, social and psychological wellbeing that PHS use to assess a person’s whole health and wellbeing through the biopsychosocial lens. Tools like Beth Frates’ paving wheel offer a structured, conceptual model for understanding whole-person health through the integration of physical, social and psychological components of health and wellbeing. Thus, guiding the way for tailored and integrative coaching strategies (Frates, 2021).

Lifestyle medicine recognizes the importance of psychosocial wellbeing, motivation, behavior change and therapeutic alliance as crucial components of health (Bodai et al., 2018; Merlo and Bachtel, 2023). Behavior change and positive emotions are powerful drivers of health and need to be leveraged with lifestyle medicine (Merlo and Bachtel, 2023). As a result, there is growing interest and research in integrating lifestyle medicine and evidence-based psychological fields like positive psychology and health psychology (Burke and Dunne, 2022; Bodai et al., 2018; Lianov and Burke, 2024).

### 2.2 Positive psychology: moving toward flourishing

Positive psychology is a scientific study of optimal human functioning, focusing on emotional, psychological, and social wellbeing (Burke, 2017). While lifestyle medicine offers strategies for improving physical and mental health, positive psychology goes beyond simply alleviating symptoms of ill-being as it promotes flourishing, which is the highest state of wellbeing (Burke et al., 2025). Additionally, while lifestyle medicine focuses on treating and eliminating the risk of chronic disease, positive psychology broadens its scope by exploring traits, states and conditions necessary for optimal functioning, regardless of an individual’s health status (Burke et al., 2022). Therefore, while distinct in focus, these two approaches are integrally connected, representing complementary pillars of positive health (Lianov and Burke, 2024). Positive psychology not only promotes physiological and psychological flourishing in conjunction with lifestyle medicine, but it also supports individuals to fully engage with lifestyle medicine practices to maximize their health and wellbeing (Lianov and Burke, 2024).

Positive psychology has developed a range of interventions for individuals and societies that build the capacity for a fulfilling and flourishing life and environments, not just interventions that decrease misery (Burke, 2017; Seligman et al., 2005; Novikova, 2024). These approaches enhance quality of life by fostering positive outcomes through hedonic (i.e., cultivating gratitude, optimism, and hope) and eudemonic interventions (i.e., improving resilience), as well as strengths-based methods such as positive diagnosis and character strengths development to foster meaning, purpose, and alignment with the authentic self (Biswas-Diener, 2010; Niemiec, 2023; Bretherton, 2025; Ungar and Jefferies, 2021). In alignment with PHS, positive psychology moves beyond the traditional focus of mental illness and instead looks at human experiences of happiness, positive emotions and flourishing. As a result, it plays a vital role in shaping the field of PHS, emphasizing the importance of wellbeing, optimism, resilience and flourishing for overall health.

Even though the objective of positive psychology is to improve wellbeing, it can also reduce stress and anxiety and improve mental states with increased optimism, resilience and overall happiness, which has been shown to improve physical health (Anusha, 2024). Positive emotions strengthen the immune system, lower the risk of cardiovascular disease, reduce inflammation and promote resilience against illnesses (Meehan, 2023). However, positive psychology has previously been criticized for lacking rigor and being self-isolating, prompting calls for a more multidisciplinary approach, a broader focus, and its application to complex situations that better reflect the current state of the world (van Zyl et al., 2024). Consequently, integrating positive psychology with lifestyle medicine within the framework of PHS may offer a complementary approach to positive health.

Lifestyle medicine and positive psychology reinforce each other through the bidirectional relationship between health-promoting behaviors and overall wellbeing. Research has shown that health-promoting behaviors can improve wellbeing and vice versa (Merlo and Bachtel, 2023; Lianov and Burke, 2024). We have demonstrated from a survey of 1,112 predominantly Irish education-based professionals that those who flourished were three times more likely to use between three and six lifestyle medicine pillars than those who were moderately well (Burke and Dunne, 2022); interestingly the same individuals who engaged in up to six of the lifestyle medicine pillars were nine times more likely to flourish compared with those who languished (Burke and Dunne, 2022). While lifestyle medicine and positive psychology provide the foundation of PHS, behavior plays an important role in creating actionable health and wellbeing changes. Health psychology can inform the behavior change and cognitive frameworks used in PHS.

### 2.3 Health psychology: building behaviors for health and wellbeing

Health psychology evolved as a field of research and practice to understand why individuals engage in certain health behaviors and how we might change these behaviors (Prestwich et al., 2017). Health psychology is a well-established interdisciplinary field that applies psychological knowledge, principles and techniques for health, illness and healthcare (Marks et al., 1998; Ogden, 2019). Health psychology looks at the pathway between behavior and health through three broad streams of research: behavioral research regarding the pathway from behavior to illness, behavioral intervention to control these pathways, and behavioral interventions to prevent and control ill health and chronic disease (Leventhal et al., 2008). Health psychology also plays a pivotal role in bringing this knowledge into action (Krantz et al., 1985). In the context of PHS, health psychology provides the necessary conceptual lens for exploring and understanding individuals’ health beliefs, cognitive factors and behaviors that may promote or inhibit behavior changes.

Many models and theories have been developed to understand the complexity of how psychological constructs can influence, predict, and change health behavior. Health psychology frameworks can be loosely grouped into theories of (1) health beliefs, such as the Health Belief Model (Rosenstock, 1966), Social Cognition Theory (Bandura, 1986) and the Transtheoretical Model of Change (Prochaska and DiClemente, 1982), and (2) theories of health behavior, like the theory of Planned Behavior, (Ajzen, 1991), Health Action Process Approach (Schwarzer, 1992) and Behavior Change Theory (Davis et al., 2015). While these seek to provide the necessary tools and techniques for guiding behavior change among individuals, it’s important to note the sheer range of theories and models for understanding the effectiveness of behavior change. Additional models including The Behavior Change Taxonomy (BCT) (Michie et al., 2013), The Theoretical Domain Framework (Cane et al., 2012), and the Capability Opportunity Motivation-Behavior (COM-B) model (Michie et al., 2014) attempt to consolidate techniques and theories into one accessible model for clinicians and researchers.

Health psychology provides a valuable conceptual lens for understanding the relationship between health and behavior, which has informed a robust foundation for disease prevention and health promotion interventions. While some health psychology interventions bring a positive, solution-focused orientation, health psychology as a discipline aligns closer to the medical definition of health as “the absence of illness” (Nelson and Simmons, 2003). Health psychology approaches are more often problem-oriented, focusing on cause and prevention of illness and disease, as well as self-management and treatment adherence (Leventhal et al., 2008; Nelson and Simmons, 2003; Ogden, 2019). Integrating health psychology with positive psychology, however, allowed for a wider, more holistic definition of health. One that moves beyond the mere reduction of pain and suffering toward promoting optimal psychological and physical functioning (Vázquez et al., 2009; Nelson and Simmons, 2003).

### 2.4 Creating the positive health paradigm

The complexity of health lends itself to multidisciplinary approaches to care and self-care that draw on a broad evidence base of complementary research, theories and frameworks (Greenhalgh and Papoutsi, 2018). PHS advocates for integrating lifestyle medicine, positive psychology, and health psychology to address the complexity of health, emphasizing that these concepts are more effective together, as each fills critical gaps in the others, creating a more comprehensive and holistic approach to health and well-being. For example, while lifestyle medicine and health psychology take a more proactive stance compared to the traditional healthcare model, they often begin with a focus on deficiency, aiming to resolve issues or restore balance. Positive psychology, however, adds a transformative dimension to PHS by shifting the focus from merely addressing problems (moving from −10 to 0) to fostering flourishing and fulfillment (progressing from 0 to +10). Likewise, while we might assume that we need to feel motivated before we can act, PHS suggest that positive emotions *and* actions build and more importantly, sustain motivation (Scott and Morgan, 2023).

However, the positive health paradigm does not merely address the complex nature of health just through a multidisciplinary theoretical approach. We propose that internal mechanisms of change such as awareness and positive orientation toward health and wellbeing are also foundational for understanding and sustaining positive health. For example, a recent controlled study of 60 college students described how self-awareness was an essential component of improved health and wellbeing outcomes (Wagani and Gaur, 2024). We use the term *meliotropism* to describe a positive orientation by any individual toward health and wellbeing, regardless of health status (Dunne et al., 2025). This type of positive orientation toward health has already been shown to modify behavior in individuals diagnosed with chronic illness (Kupcewicz et al., 2019). We suggest that this positive orientation can be accomplished despite a medical diagnosis, the aging process, socio-economic status, or the nature of political and physical environments. By cultivating *meliotropism*, individuals might shift from passive recipients of care to active participants in their health journey, reinforcing the principles of positive health (Dunne et al., 2025; Ryff and Singer, 2008).

This paper has highlighted the necessary interplay between health psychology’s conceptual understanding of behavior changes and health belief, positive psychology’s promotion of eudemonic and hedonic wellbeing for flourishing, lifestyle medicine’s evidence-based strategies, and individuals’ internal mechanisms of change for fostering healthier lifestyles and reducing chronic disease risk. Together, they form a comprehensive framework of holistic positive health. Building on these theories, PHS integrates medical and psychological approaches to health and wellbeing. To translate this emerging field into practice, coaching has been identified as a valuable tool for applying these concepts in real-world settings.

## 3 Coaching: translating concepts into practice

Coaching has emerged as a pivotal tool for delivering health promotion, disease prevention, and wellbeing interventions and supporting individuals to achieve their goals (Casadei et al., 2019; Butterworth et al., 2007; Dejonghe et al., 2017). Coaching is a professional relationship where coaches partner with clients in a thought-provoking and creative process that inspires them to unlock previously untapped resources available to them and maximize their personal potential (International Coaching Federation, n.d.). Coaches work with clients to facilitate experiential learning, improved functioning, and performance, generally while working toward specific goals (Davison and Gasiorowski, 2006). Each contributing field of PHS has recognized the value of coaching as a method of applying its concepts to practice (Burke, 2017; Reynolds et al., 2018; Conn and Curtain, 2019; Olsen, 2014). Integrating coaching with rigorous fields of health and wellbeing creates a research-informed, holistic coaching approach that can work alongside traditional healthcare (van Nieuwerburgh and Knight, 2023).

Health coaching has emerged as a valuable health-focused coaching approach, which consists of health education and promotion to enhance the health and wellbeing of clients and support the achievement of health-related goals (Olsen, 2014). It is largely a behavioral health intervention, focused on lifestyle-related behavioral actions and intentions, readiness to change, motivation, and self-efficacy with the intention of reducing health risks, improving self-management of NCDs and increasing health-related quality of life (Butterworth et al., 2007; Kivelä et al., 2014). Motivational interviewing is the most rigorously studied intervention of health coaching, which is a counseling style designed to elicit behavior change by exploring and resolving ambivalence and moving through the stages of change (Butterworth et al., 2007; Olsen and Nesbitt, 2010). This coaching approach has become popular for disease management, as it addresses multiple behaviors, health risks, and self-management, particularly for smoking cessation, T2DM, and cardiovascular disease (Butterworth et al., 2007; Olsen and Nesbitt, 2010). Subsequently, health coaching has been incorporated into health management programs in a variety of settings, both clinical and non-clinical settings such as workplaces and communities. While health coaching effectively addresses the behavioral focus and lifestyle medicine guidelines central to PHS, it falls short of incorporating core concepts of positive psychology and the overarching goal of bridging medical and psychological approaches to health and wellbeing, as envisioned in PHS. To fully capture the essence of PHS, innovative approaches like positive psychology coaching (PPC) and dialogical coaching must be explored and integrated.

### 3.1 Positive psychology coaching

Positive psychology is a foundational pillar of PHC, thus any professional interested in practicing PHS must be knowledgeable in Positive Psychology Coaching (PPC) and its fields of knowledge relating to wellbeing, positive emotions, flow, relationships, strengths, positive traits, gratitude, motivation, self-determination, creativity, resilience, self-efficacy, hope, perceived control and meaning (Van Zyl et al., 2020). The in-depth knowledge of PP is essential for three main reasons. First, it allows coaches to spot their clients’ internal processes, such as optimism, hope, and grit, which are often challenging to identify without developing knowledge and skills. This will enable them to refocus their clients’ attention from deficits to strengths to facilitate positive outcomes. For example, when a client wants to quit smoking but feels helpless due to repeated failed attempts, a coach trained in PP can recognize pessimistic thinking style or helplessness and consequently guide clients using one of the more relevant pathways. Second, a firm grounding in PP allows coaches to apply evidence-based tools to maximize clients’ potential. Combined with lifestyle medicine strategies (see Lianov and Burke, 2024 for a review), these can significantly enhance physical and psychological outcomes. For example, instead of addressing stress through relaxation or other stress management techniques, coaches might explore clients’ *stress mindsets*, believing that stress can be enhanced (Crum et al., 2017), thereby unlocking more adaptive coping mechanisms for coping with stress without directly addressing the need to reduce stressors. Third, PP provides an important framework for supporting long-term, values-based behavior change. By helping clients connect their health goals to a deeper sense of meaning and purpose, coaches can support clients in tapping into their intrinsic motivation and making their health journey more meaningful. They would not be possible to do so without their in-depth knowledge of PP, which informs the questions they ask.

The PPC has been defined as “a managed conversational process that supports people to achieve meaningful goals in a way that enhances their wellbeing” (Biswas-Diener and van Nieuwerburgh, 2021, p. 315). The Conceptual Framework of Positive Psychology Coaching provides a comprehensive approach to PPC practice (Burke, 2017). According to the PPC model, practitioners ought to have in-depth knowledge of positive psychology to practice it. Most PPC models incorporate components such as developing strengths, setting up realistic goals, empowerment, and creating relationships. Through the utilization of PPC, positive health practitioners focus on human strengths, positive resources available to clients, and positive habits already built by clients to achieve optimal functioning and reach their goals. Likewise, positive psychology interventions (PPIs) and positive measures are used to build positive effects in clients like optimism, hope, gratitude and locus of control. The model has been applied in practice and shown positive outcomes when working with clients and as a structure for training coaches (Lucey and van Nieuwerburgh, 2021; Toneatto et al., 2024).

In PHS, PPC balances hedonic and eudemonic wellbeing to create sustainable and holistic wellbeing. Hedonic-focused PPIs, such as those involving immediate engagement in pleasurable activities and cultivating gratitude, are valuable for fostering positive emotions. However, to build long-term, sustainable wellbeing, eudemonic approaches rooted in sustained personal growth and purpose are essential. Eudemonic theories such as Self-Determination Theory, Resilience Building, and the Strengths-Based approach, as well as frameworks like Positive Diagnosis, complement hedonic interventions by encouraging individuals to explore meaning, develop personal strengths, and align with their authentic selves. Together, these approaches provide a holistic framework for promoting both immediate and enduring wellbeing.

### 3.2 Dialogical coaching

PHS is an interdisciplinary field which integrates medical and psychological fields of knowledge into the newly emerging domain of positive health. We know that these fields of knowledge, while distinct in focus are closely interconnected and can serve complementary roles within the PHS framework (Lianov and Burke, 2024). Likewise, medical and psychological approaches to delivering these concepts to patients or clients can be distinct. Medical practitioners often take a directive approach to delivering health promotion interventions, while psychology and coaching practitioners generally take a facilitative, person-centered approach to health and wellbeing (Rogers, 2000; Joseph and Murphy, 2013; Davison and Gasiorowski, 2006; Ramdurai, 2020).

Dialogical coaching blends the traditional facilitative approach to communication found in coaching and positive psychology with the knowledge-sharing approach evident in healthcare and lifestyle medicine (van Nieuwerburgh and Knight, 2023). This new dialogical framework helps empower and inform patients and clients, balancing inquiry with knowledge-sharing to adapt to the client’s needs. Through dialogical conversations, clients and patients are viewed as experts in their own lives and are supported, rather than directed, in setting goals and developing their own solutions. At the same time, coaches strategically share knowledge to enhance individuals’ skills and understanding, empowering them to take control of their health and wellbeing (van Nieuwerburgh and Knight, 2023). For example, positive health practitioners apply a dialogical approach in which evidence-based information on the six pillars of lifestyle medicine or PPIs can be shared with clients and patients about best health and wellbeing practices. While sharing knowledge, clients remain at the center of the conversation. Information is shared strategically only with the consent of the clients, to support clients’ knowledge and skill development and to build autonomy and control over their health and wellbeing. Outside of information sharing, coaches default to a facilitative, client-centered approach (i.e., for goal setting or discovery).

The integration of health coaching, PPC, and dialogical coaching paves the way for a new coaching approach: Positive Health Coaching (PHC). PHC represents a comprehensive approach that bridges the directive and facilitative strategies of coaching, blending evidence-based interventions with a holistic focus on physical, psychological, and emotional wellbeing. By incorporating the strengths of Health Coaching, PPC, and Dialogical Coaching, PHC aligns with the broader vision of PHS, empowering individuals to move beyond functioning to flourishing physically and mentally.

## 4 Positive health coaching

**Figure 3:**
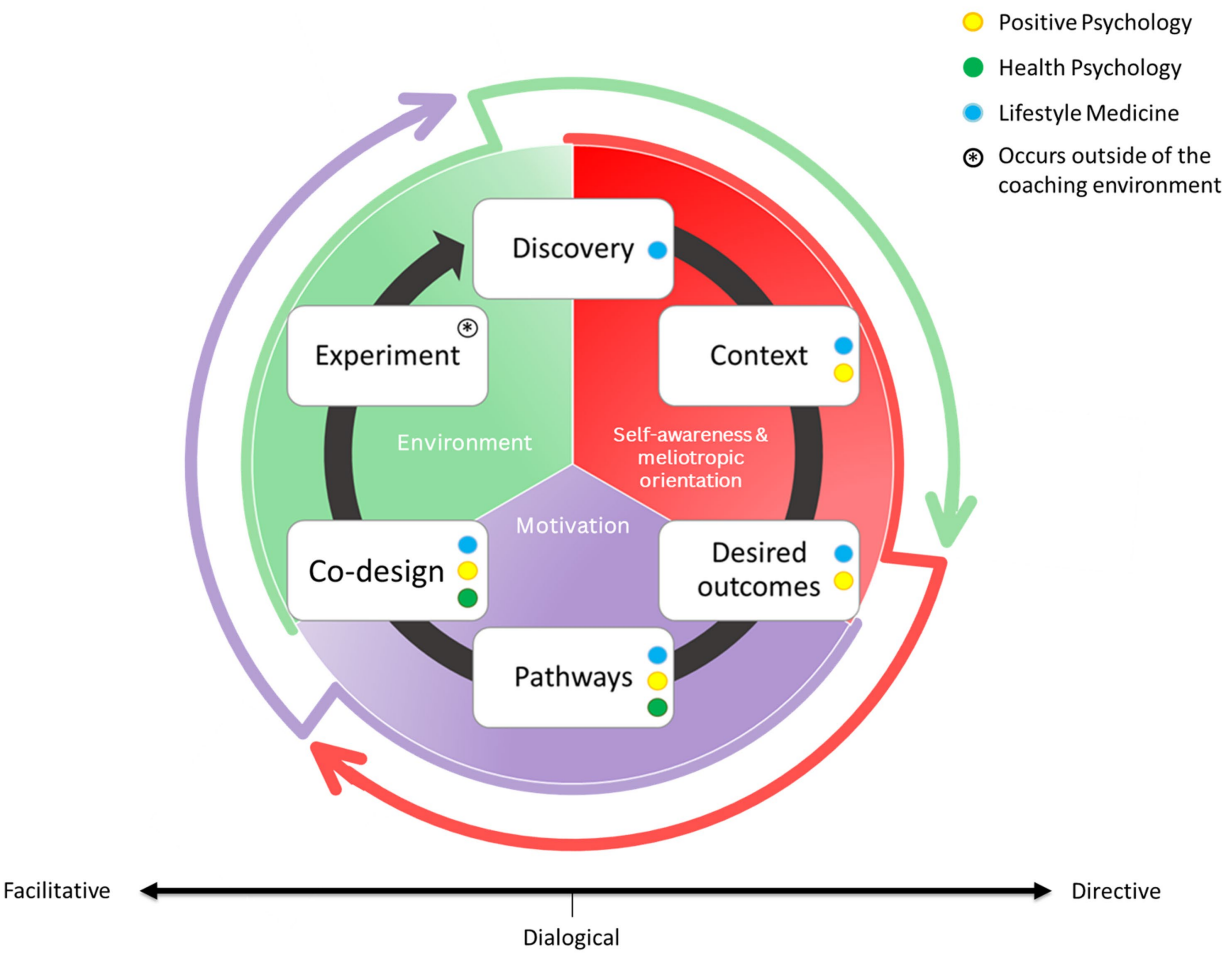
Positive health coaching conversational framework. This new PHC model is modified and adapted with permission from van Nieuwerburgh and Knight (2023), with consent from the authors. The PHC relationship between client and coach is structured around six dynamic, interlinked stages: discovery, context, desired outcomes, pathways, Co-design, and experiment. This conversational model is based on a foundation of three overarching determinants of health and wellbeing that influence the process: self-awareness and meliotropic orientation (red), motivation (purple), and environment that includes elements of the social, systemic, and natural worlds (green). The color-coded dots represent the theoretical and evidence-based pathways that can be used to support the client along their coaching journey: positive psychology (yellow), health psychology (green), and lifestyle medicine (blue). The cyclical flow reflects a continuous, client-led process of reflection, action, and adaptation in pursuit of health and wellbeing.

The PHC is essentially an intervention designed to support people to set health and well-being-related goals and then work toward them. At its core, it is a dedicated conversational framework (Figure 3), and coaching skills adapted from health coaching, PPC and dialogical coaching (van Nieuwerburgh and Knight, 2023). The conversational framework provides a structured yet flexible guideline for PHC, illustrating how the coaching dialog unfolds across three internal stages of change: self-awareness and meliotropic orientation, motivation, and environment. It also shows how PHC integrates lifestyle medicine, positive psychology, and health psychology throughout the coaching conversation (Figure 3).

### 4.1 Self-awareness and meliotropic orientation

The first two stages of the conversation, *discovery* and *context*, focus on building self-awareness and cultivating meliotropic orientation. Here, the coach uses the six pillars of lifestyle medicine to guide an explorative discussion with clients about their overall health and wellbeing. Effective communication skills (i.e., active listening, reflection, summarizing, validating) and strong therapeutic alliance is crucial to this phase of the conversation. As the client and coach move into context, positive psychology becomes central. Rather than focusing primarily on deficits or challenges, the coach supports the client in identifying personal strengths, values, and current and past successes. This builds a sense of accomplishment and confidence early in the coaching conversation. It is important to note that positive psychology continues to play a central role throughout the framework, as it is vital to build strengths and resources within clients. Clients are also encouraged to reflect on their wider life context (i.e., work, family, community, and environment) and how these factors support or hinder their positive health journey. Together, discovery and context build a robust understanding of the client’s lived experience and internal resources, creating a strong foundation of self-awareness and meliotropic orientation which continues to grow throughout the conversation.

### 4.2 Motivation

As the conversation progresses, the client and coach move into the motivation phase, which consists of *desired outcomes* and *pathways*. This future-oriented stage involves exploring the client’s motivations and aspirations to create a vision for the future (desired outcomes). This vision gives direction and meaning to the clients’ positive health journey, helping to build motivation. As direction and motivation build, the focus turns to *pathways (usually evidence-based lifestyle medicine and positive psychology-based guidelines, approaches and interventions),* where the coach and client co-create multiple routes which will bring them closer to their vision for the future. Health psychology is introduced during this phase, drawing on behavior change models and evidence-based coaching techniques to support the development of self-efficacy, commitment, and readiness to change. However, the dialogical approach to PHC is also central in this phase. This is what sets PHC apart from other coaching frameworks. Adopting a dialogical approach, coaches can share research-informed or evidence-based practices that might be helpful to the client. Guidelines on lifestyle medicine-based pillars such as promoting quality sleep can be provided here, for example. This is particularly useful when clients lack confidence or health knowledge. However, it is important to note that any information shared by the coach to the client in PHC is shared in a way that maintains the client’s autonomy and agency at all times. This remains a non-prescriptive conversation, and clients are encouraged to be creative and unbounded during this stage.

### 4.3 Environment

Once pathways toward the personal positive health vision have been identified, the conversation moves into the environment phase (*Co-design* and *experiment*). This focuses on bringing these pathways and ideas into action within the clients’ environment. During *Co-design*, the coach and client collaborate to develop plans for action in a way that is most helpful for the client. These plans are clear, specific, attainable, buildable, and intentionally shaped to align with the client’s environment to increase the feasibility of new behaviors. Finally, the *experiment* stage of the framework involves clients implementing these changes in their environment outside of the coaching conversation. However, clients may remain in contact with the coach for support if challenges arise. At the next session, the cycle resumes with a reflective *discovery* stage, now focusing on review. By returning to the coaching session through self-awareness and meliotropic orientation (phase 1), it strengthens client insight, enabling further growth and understanding.

Although not explicitly captured in the PHC conversational framework, the person at the center of the conversation remains its most vital element. While the framework guides coaches and practitioners through different phases of PHC that build awareness, motivation, and action, PHC is fundamentally rooted in a human-centered ethos. It is a patient-centered, non-prescriptive, and autonomy-supporting approach that prioritizes co-design and collaboration with individuals who are ready to engage in change. PHC is not about persuading individuals to make changes. It is about respecting each client’s readiness, autonomy, and personal vision for positive health. Effective PHC also requires genuine respect for the client’s lived experience, values, expertise and knowledge, empowering them to take greater responsibility and ownership of their health and wellbeing.

By leveraging therapeutic alliances, conversational skills, PPC, and an integrated dialogical approach, PHC combines the strengths of both coaching and healthcare disciplines into a unified framework. However, PHC is not simply a collection of these domains. The key aspect of PHC is the balance between seemingly diverging fields of lifestyle medicine, positive psychology, and health psychology. It is defined by its ability to interweave and balance them in a dynamic, client-centered process. PHC practitioners should have the ability to flexibly draw from lifestyle medicine, positive psychology, and health psychology to adapt to the client’s needs and context. Dropping or overemphasizing one area diminishes the holistic essence of PHC. For instance, a practitioner exclusively offering lifestyle advice and lifestyle prescriptions is practicing lifestyle medicine, not PHC. Similarly, focusing solely on facilitating self-discovery and cultivating individual strengths, gratitude and growth, as in PPC, is not PHC. Rather, PHC involves the synergistic blending of information about lifestyle medicine with growth-oriented, person-centered dialog, fostering whole-person health and wellbeing. This is achieved through knowledge of lifestyle medicine, PP, and health psychology, and techniques and strategies drawn from PPC and dialogical coaching.

### 4.4 PHC: potential and pathways

The application of PHS through PHC in current healthcare pathways, both clinical and non-clinical, represents a promising avenue for healthcare systems to support individuals’ health beyond merely avoiding illness. This aligns with the World Health Organisation’s definition of health as “a state of complete physical, mental, and social well-being and not merely the absence of disease or infirmity” (World Health Organisation, 1995). For the worried well, PHC has the potential to transition this population to a state of flourishing, empowering individuals to take control of their health by providing motivational support and evidence-based tools for sustainable life changes. This can significantly lower the risk of developing non-communicable diseases (NCDs) and enhance overall mental and physical well-being. Likewise, in managing chronic diseases, PHC offers crucial support by assisting patients in handling their conditions and associated comorbidities. Aligning with the concepts of patient-centered care (PCC) (Louw et al., 2017; Moody et al., 2018), PHC reduces the fragmentation of care by integrating support throughout different stages of disease progression. Co-designing strategies with patients can further improve their quality of life, aid in managing existing conditions, and help prevent new comorbidities. Through continuous, coordinated care, patients can receive holistic and comprehensive treatment that addresses not only NCDs but also the various comorbidities associated with these conditions.

Various health professionals can utilize the PHC model in a variety of settings to have maximal reach to the general population. Because of its foundations in healthcare, psychology and coaching, PHC can be delivered by trained healthcare professionals during routine appointments with patients or by trained psychologists and coaches as an independent health appointment that supports recommendations by health professionals. Limited evidence exists on the impact of team coaching (Jones, 2022). However, the available studies, though varied in nature, suggest a positive impact (Traylor et al., 2020). Future research should examine the effects of PHC when implemented as a team-based intervention. As a result, PHC has the potential for adaptation across various healthcare systems and into health insurance programs to be delivered by healthcare professionals, psychologists, and coaches alike. While this is still a newly emerging approach, research is starting to show the impact of PHC on supporting internal mechanisms of change (i.e., awareness and meliotropic orientation toward positive health), as well as positive health and wellbeing outcomes (O'Donovan et al., 2024; Dunne and Loughnane, 2023; Loughnane et al., 2025). However, as PHC continues to evolve, appropriate training, regulation, and governance are essential to ensure its safe, ethical, and competent practice. At a minimum, researchers and practitioners should receive training from reputable institutions such as the Centre for Positive Health Sciences, and seek accreditation from relevant regulatory bodies, such as the European Mentorship and Coaching Council (EMCC). Ongoing supervision from a qualified health or coaching psychologist is also important, particularly as the practice and application of PHC expand and are disseminated more widely.

## 5 Conclusion

Positive health coaching (PHC) represents a transformative and adaptable approach to addressing the gaps in current healthcare by integrating the evidence-based disciplines of positive psychology, health psychology and lifestyle medicine. Grounded in positive health sciences (PHS), PHC mobilizes key fields such as lifestyle medicine, positive psychology, and health psychology to create a holistic, proactive framework that empowers individuals to flourish physically and mentally, regardless of their health status. Here, we provide a conceptual analysis of these disciplines, highlighting how these seemingly distinct fields contribute to a holistic and proactive approach to health and well-being. Similarly, by highlighting their shared principles, we describe how these fields complement each other—reinforcing strengths while addressing limitations. Additionally, PHC’s blend of approaches, including positive psychology and dialogical coaching, broadens its relevance across diverse settings.

Ultimately, this conceptual analysis provides the foundation for how PHC as a multidisciplinary, evidence-based model was developed and its potential benefits for addressing the complexity of health and wellbeing. As PHC continues to develop, further research is needed to refine how it can be integrated and adapted across different environments and who it benefits most. Likewise, while research is emerging showing the positive impact PHC has on health and wellbeing, the long-term impact of PHC must be examined. Finally, the provision of high-quality training in PHC that is accessible to healthcare professionals, psychologists, and coaches is of utmost importance to protect the integrity and rigor of PHC.
